# miR-193a Directly Targets PSEN1 and Inhibits Gastric Cancer Cell Growth, the Activation of PI3K/Akt Signaling Pathway, and the Epithelial-to-Mesenchymal Transition

**DOI:** 10.1155/2021/2804478

**Published:** 2021-07-14

**Authors:** Xuemei Pan, Ting Zhao, Saisai Mu, Shouchuan Li

**Affiliations:** ^1^General Surgery Ward I, Qingdao Hospital of Traditional Chinese Medicine, Qingdao Hiser Hospital, Qingdao 266000, China; ^2^General Surgery Ward III, Qingdao Hospital of Traditional Chinese Medicine, Qingdao Hiser Hospital, Qingdao 266000, China

## Abstract

**Background:**

Gastric cancer, a kind of gastrointestinal malignancy, is the second type of leading death cancer. miR-193a is a key tumor suppressor in several diseases. PSEN1 is mainly related to Alzheimer's disease and may be involved in the cleavage of the Notch receptor. *Material and Methods*. RT-PCR and western blot were applied to evaluate miR-193a and the expression level of PSEN1. Luciferase reporter assay was applied to verify whether PSEN1 was a target of miR-193a. The Kaplan–Meier method was employed to calculate the 5-year overall survival of gastric cancer patients.

**Results:**

miR-193a was downregulated in gastric cancer tissues and cell lines, and downregulation of miR-193a predicted poor 5-year overall survival of gastric cancer. miR-193a inhibited the proliferation and the activation of the PI3K/AKT signaling pathway in gastric cancer cells. miR-193a inhibited gastric cancer tumor growth in vivo. miR-193a impaired cell invasion and epithelial-to-mesenchymal transition (EMT) in HGC-27 cells. In addition, PSEN1 was a direct target of miR-193a and PSEN1 reversed partial functions of miR-193a in cell proliferation and invasion.

**Conclusion:**

miR-193a prominently decreased the proliferation, invasion, and activation of the PI3K/Akt signaling pathway and the abilities of epithelial-to-mesenchymal transition in gastric cancer cells. The newly identified miR-193a/PSEN1 axis provides novel insight into the pathogenesis of gastric cancer.

## 1. Introduction

Gastric cancer (GC), the second leading death cancer, is a kind of gastrointestinal malignancy [1]. Lamentably, gastric cancers patients at an early stage are difficult to diagnose due to no specific symptoms [2]. Thus, exploring new biomarkers for the early diagnosis and treatment of gastric cancer is urgent.

Micro-RNAs (miRNAs), short noncoding RNAs, could cause mRNA degradation or translational inhibition through binding to target mRNAs 3′-UTR at the posttranscriptional level [3, 4]. Recently, accumulating evidence indicated that miRNAs play a vital role in tumor progression in gastric cancer, including proliferation, metastasis, and apoptosis [5–7]. miR-193a acted as a critical tumor suppressor in various cancers, including colon cancer, acute myeloid leukemia, bladder cancer, and malignant pleural mesothelioma [8–11]. In non-small cell lung cancer, Yu et al. illuminated that miR-193a suppressed cell migration, invasion, and EMT [12]. Similarly, Fang et al. demonstrated that miR-193a inhibited cell proliferation and metastasis in pancreatic cancer [13]. Therefore, we strongly believe miR-193a inhibited cell proliferation and invasion in gastric cancer.

Presenilin-1 (PSEN1), a primary component of the *γ*-secretase complex, is mainly related to Alzheimer's disease and may be involved in the cleavage of the Notch receptor [14, 15]. The mutation of PSEN1 was associated with spasticity and parkinsonism in the dominant Alzheimer's family [16]. Zhao et al. indicated that PSEN1 overexpression results in attenuated phagocytic uptake of A*β* by microglia and regulated intracellular trafficking and pathophysiological function in myeloid cells [17]. In addition, Meng et al. illuminated that PSEN1 was a target gene of miR-193a and miR-193a regulated chemoradiation resistance through PSEN1 in oesophageal cancer [18].

In this present study, we aimed to investigate the biological effect of miR-193a on gastric cancer. To translate these findings to clinical practice, it is essential to explore the molecular mechanism of miR-193a in the development of gastric cancer.

## 2. Materials and Methods

### 2.1. Patients and Tissue Samples

We selected 50 patients that underwent an operation at Qingdao Hospital of Traditional Chinese Medicine, Qingdao, China, and obtained 50 pairs of gastric cancer tissues and corresponding peritumoral normal tissues. The specimens were immediately frozen in liquid nitrogen and followed storage at −80°C. Informed consent was obtained from each patient and this investigation was approved by the ethics committee of the Qingdao Hospital of Traditional Chinese Medicine, Qingdao, China.

### 2.2. Cell Lines and Culture Conditions

We obtained two human gastric cancer cells HGC-27 and MGC-803 and a normal gastric epithelial cell GES-1 from American Type Culture Collection (ATCC, Rockville, MD, USA). RPMI-1640 (Gibco-BRL; Rockville, MD, USA) medium contained with 10% FBS (Haoyang Biological, Tianjin, China) were conducted to culture the cells at 37°C with 5% CO_2_.

### 2.3. Plasmid Construction and Transfection

We purchased the miR-193a mimic and miR-193a inhibitor oligo fragments from Gene-Pharma (Shanghai, China), which was conducted to upregulate or downregulate miR-193a. HGC-27 cells were seeded in a 6-well plate, and the transfection was performed using Lipofectamine 2000 (Invitrogen, Carlsbad, USA) reagent as described previously [19]. In brief, the Opti-MEM/reduced serum medium (Thermo Fisher Scientific, Shanghai, China) was employed to dilute the Lipofectamine 2000 reagent and the oligo fragments, respectively. After mixing the two solutions, the mixture was added to the cells. For the transiently transfected cells, the cells were harvest after 48 h. On the contrary, for the cells with stable transfection, we applied Geneticin (G418, Thermo Scientific, Shanghai, China) to select.

### 2.4. RNA Extraction and Quantitative Real-Time PCR

TRIzol reagent (Invitrogen) and mirVana miRNA Isolation Kit (Thermo, Shanghai, China) were employed to extract the total RNAs and total miRNAs, respectively. The first cDNA chains of mRNA and miRNA were synthesized by the Prime Script RT Reagent Kit (Takara Biotechnology Co., Ltd., Dalian, China) and the miRNA Reverse Transcription Kit (Life Technologies, Foster, CA, USA), respectively. The qPCR was performed by using the SYBR Premix Ex Taq (Takara) and MystiCq microRNA qPCR Assay Primer (Sigma, Missouri, USA) on Applied Biosystems StepOnePlus™ Real-Time PCR System. The normalization for miR-193a and PIK3CG was used as U6 and GAPDH, respectively. The primers were miR-193a F: 5′-ACTGGCCTACAAAGTCCCAGT-3′, R: 5′-GTGCAGGGTCCGAGGT-3'; U6 F: 5′-CTTCGGCAGCACATATAC-3′, R: 5′-GAACGCTTCACGAATTTGC-3'; PSEN1 F: 5′-TATGGCAGAAGGAGACCCG-3′, R: 5′-CCATTCCTCACTGAACCCG-3'; GAPDH F: 5′-AAGGTGAAGGTCGGAGTCAA-3′, R: 5′-AATGAAGGGGTCATTGATGG-3'.

### 2.5. Western Blot Analysis

RIPA Lysis Buffer (Sigma, USA) containing 10% PMSF (Sigma, USA) was conducted to lyse the HGC-27 cells on ice for 30 min. We centrifuged the protein solving liquid at 4°C for 20 min with 12,000  × g speeds and then collected the supernatants. We separated the proteins by electrophoresis utilized 10% SDS-PAGE and then transferred the blots on a PVDF membrane (Millipore, USA). After blocking by 5% fat-free milk in TBST buffer at room temperature for 1 h, the blots were incubated with the primary antibodies at 4°C overnight. The membrane was incubated by primary antibodies, including PSEN1 (1 : 1000; Abcam, Cambridge, USA), EMT proteins (E-cadherin (1 : 1000; Abcam), and N-cadherin (1 : 1000; Abcam) and the proteins on the PI3K pathway (p-PI3K (1 : 1000, Cell Signaling, San Jose, CA, USA), PI3K (1 : 1000, Cell Signaling), p-AKT (1 : 1000, Cell Signaling), AKT (1 : 1000, Cell Signaling)), and GAPDH (1 : 1000, Santa Cruz, Shanghai, China). Subsequently, we incubated the membranes by anti-rabbit or mouse HRP-conjugated secondary antibody for 2 h at room temperature. In the end, Enhanced Chemiluminescence (ECL, Pharmacia Biotech, Arlington, USA) was employed to evaluate the signals.

### 2.6. CCK-8 Assay

The cell proliferative ability was conducted by Cell Counting Kit-8 (CCK8, Dojindo, Japan) [19]. HGC-27 cells were seeded in a 96-well plate and cultured for 24 h, 48 h, 72 h, or 96 h at 37°C incubator. After being cultured, 10 *μ*l of CCK8 solutions was added to each well and cultured at 37°C for 1 h; then, the absorbance was assessed at 450 nm using a microplate reader (BioTek, Winooski, USA).

### 2.7. Transwell Assays

The cell invasive ability was measured by transwell insert (8 *μ*m membrane, Corning, Cambridge, MA) with matrigel (BD Biosciences) covered [19]. The inserts were put in a 24-well plate to form the upper and lower chambers. The 200 *μ*l HGC-27 cells' suspension was added in the upper chamber, which was suspended by RPMI-1640 medium without PBS. Meanwhile, the lower chamber was filled with a 500 *μ*l RPMI-1640 medium containing 15% FBS. After incubating the cells at 37°C for 24 h, the noninvasive cells on the upper surface were removed using a cotton swab. For the invaded cells, they were fixed and then stained using 4% paraformaldehyde and 10% crystal violet in sequence; and then, the cells were counted under the microscope (Olympus Corporation, Tokyo, Japan).

### 2.8. Dual-Luciferase Reporter Assay

TargetScan predicted PSEN1 was a target gene of miR-193a, and the binding site was located at 697–703 on PSEN1 mRNA 3′-UTR. To verify whether miR-193a directly binds to PIK3CG mRNA 3′-UTR, the binding sequences were mutated from GGCCAGU to CCGGUCA and inserted in pmirGLO luciferase reporter vector. The HGC-27 cells were cotransfected with miR-193a mimic and the wild-type 3′-UTR or the mutant 3′-UTR of PSEN1. We utilized the dual-luciferase reporter assay system (Promega) to evaluate the firefly luciferase activity with Renilla luciferase activity as the normalization.

### 2.9. Tumor Xenograft Model in Nude Mice

The 4-week-old nude mice were purchased from Charles River Laboratories (Beijing, China). The nude mice were randomly classified into two groups, miR-193a mimic miR-193a control groups, with two mice in each group. HGC-27 cells were inoculated into the nude mice through subcutaneous injection. The mice were raised in the same environment and were observed every week. We measured and recorded the tumor lengths and widths and then calculated the tumor volume every three days. All animal experiments were performed in the animal laboratory center of Qingdao Hospital of Traditional Chinese Medicine and approved by the Care and Use Committee of Qingdao Hospital of Traditional Chinese Medicine.

### 2.10. Statistical Analysis

All the data were analyzed by SPSS statistical software version 16.0 (SPSS, Chicago, IL, USA), which were presented as the mean ± standard deviation. The significance between two groups and multiple groups was compared by a two-tailed Student's *t*-test and one-way analysis of variance followed by an LSD test. *P* value < 0.05 was found to be statistically significant.

## 3. Results

### 3.1. Downregulation of miR-193a Predicts Poor Prognosis of Gastric Cancer

**Figure 1:**
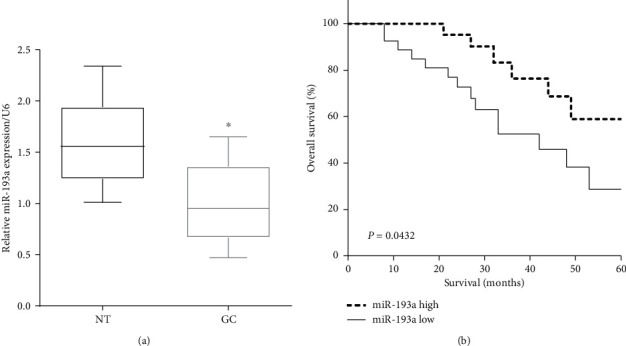
Downregulation of miR-193a predicts poor prognosis of gastric cancer. (a) miR-193a expression was low in GC tissues versus corresponding peritumoral normal tissues. (b) Low expression of miR-193a predicted poor prognosis of gastric cancer patients. ^*∗*^*P* < 0.05.

We evaluated the mRNA levels of miR-193a in 50 pairs of gastric cancer and peritumoral normal tissues. As expected, the expression of miR-193a was lower in GC tissues than that in the corresponding peritumoral normal tissues (*P* < 0.05) (Figure 1(a)). According to the median level of miR-193a, patients were divided into a low expression group and a high expression group [20]. Results showed that high expression of miR-193a was associated with poor prognosis in gastric cancer (*P* < 0.05) (Figure 1(b)).

### 3.2. miR-193a Suppresses Cell Proliferation and Inhibits the Activation of PI3K/Akt Signaling Pathway in Gastric Cancer Cells

**Figure 2:**
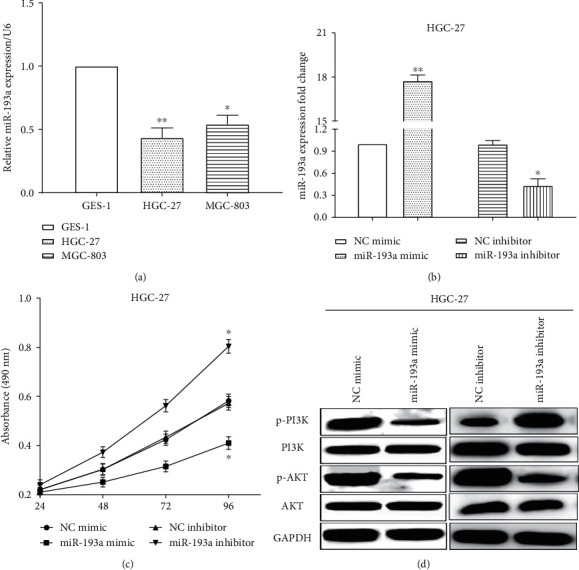
miR-193a suppresses the activation of PI3K/AKT pathway in gastric cancer cells. (a) miR-193a expression was low in GES-1 cells versus HGC-27 and MGC-803 cells. (b) The miR-193a mimic and miR-193a inhibitor were conducted to upregulate or downregulate miR-193a in HGC-27 cells measured by RT-qPCR. (c) CCK8 assay indicated miR-193a mimic suppressed cell proliferation, while miR-193a inhibitor promoted cell proliferative ability in HGC-27 cells. (d) miR-193a inhibited the activation of PI3K/AKT pathway. ^*∗*^*P* < 0.05; ^*∗∗*^*P* < 0.01.

We calculated miR-193a expression in two gastric cancer cells (HGC-27 and MGC-803) and a normal epithelial cell GES-1. The same as the tissues, miR-193a was low expressed in GES-1 cells versus HGC-27 (*P* < 0.01) and MGC-803 (*P* < 0.05) cells (Figure 2(a)). To detect the significant roles of miR-193a, miR-193a mimic and miR-193a inhibitor were conducted to upregulate (*P* < 0.01) or downregulate (*P* < 0.05) miR-193a in HGC-27 cells measured by RT-qPCR (Figure 2(b)).

CCK8 assay indicated that miR-193a mimic suppressed (*P* < 0.05) cell proliferation, while miR-193a inhibitor promoted (*P* < 0.05) cell proliferative ability in HGC-27 cells (Figure 2(c)). Western blot assay was conducted to assess the expression of PI3K pathway-associated proteins in HGC-27 cells. We discovered that miR-193a overexpression suppressed p-PI3K and p-AKT expression in HGC-27 cells, whereas miR-193a inhibitor enhanced the expression of p-PI3K and p-AKT, which elucidated that miR-193a inhibited the activation of PI3K/Akt signaling pathway (Figure 2(d)).

### 3.3. miR-193a Suppresses the Gastric Cancer Growth In Vivo

**Figure 3:**
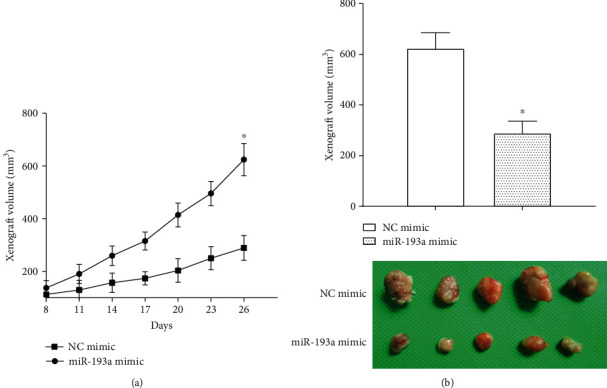
miR-193a suppresses the xenograft growth in vivo. (a) miR-193a overexpression inhibited gastric cancer xenograft growth. (b) The tumor volume of cells overexpressed miR-193a was smaller than the control group. ^*∗*^*P* < 0.05.

HGC-27 cells stably transfected with miR-193a mimic or control plasmid were applied to inject into the nude mice at subcutaneous. The xenograft tumors volumes were calculated every 3 days and the group of transfecting miR-193a mimic had a slower growth rate than the control group (Figure 3(a)). After 26 days of culture, the nude mice were dissected and the tumor volume was calculated and recorded. Furthermore, we discovered that the tumor volumes in miR-193a overexpression group were smaller than those in control group, which indicated that overexpressed miR-193a inhibited gastric cancer growth *in vivo* (*P* < 0.05) (Figure 3(b)).

### 3.4. miR-193a Inhibits Cell Invasion and the EMT in HGC-27 Cells

**Figure 4:**
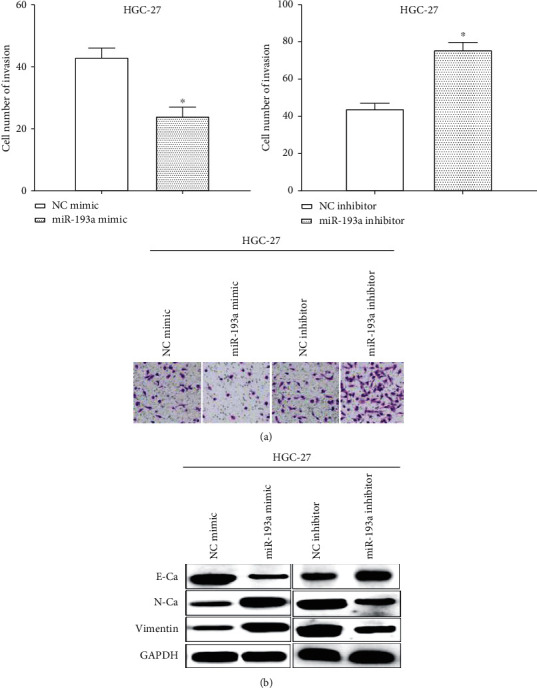
miR-193a suppresses cell invasion and EMT in gastric cancer cells. (a) Transwell assay elucidated that miR-193a inhibited the abilities of invasion in gastric cancer cell HGC-27. (b) miR-193a inhibited the EMT in HGC-27 cells. ^*∗*^*P* < 0.05.

Transwell assay elucidated that miR-193a mimic suppressed (*P* < 0.05) cell invasive ability whereas miR-193a inhibitor enhanced cell invasive ability (*P* < 0.05) (Figure 4(a)). All the findings revealed miR-193a inhibited the abilities of proliferation and invasion in gastric cancer cell HGC-27. The EMT ability was evaluated by detecting the expression of EMT-associated proteins by western blot. As we expected, miR-193a mimic suppressed the expression of N-cadherin, while improving the expression of E-cadherin in HGC-27 cells. On the contrary, miR-193a inhibitor enhanced the expression of N-cadherin and decreased E-cadherin expression, which suggested that miR-193a inhibited cell EMT through PSEN1 (Figure 4(b)).

### 3.5. miR-193a Regulates the Expression of PSEN1 through Directly Binding to 3′-UTR of PSEN1 mRNA

**Figure 5:**
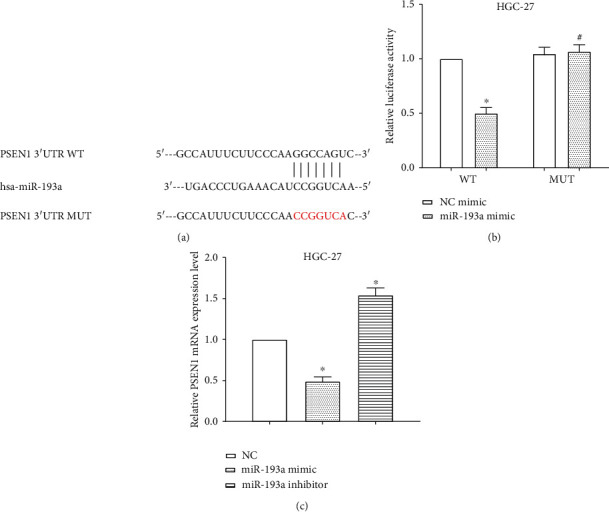
miR-193a regulates PSEN1 expression through binding to PSEN1 mRNA 3′-UTR. (a) PSEN1 was a target gene of miR-193a, and the binding site was located at the 3′-UTR of PSEN1 mRNA. (b) Luciferase reporter assay elucidated miR-193a and decreased the luciferase activity of HGC-27 cells transfected with wild-type PSEN1 3′-UTR, while it did not alter the luciferase activity of cells transfected with mutant PSEN1 3′-UTR. (c) Overexpression of miR-193a inhibited the mRNA level of PSEN1, while knockdown of miR-193a enhanced the expression of PSEN1 in HGC-27 cells. ^*∗*^*P* < 0.05; ^#^*P* < 0.05.

TargetScan predicted one of the target genes of miR-193a was PSEN1, and the binding site was located at 697–703 on PSEN1 mRNA 3′-UTR. To verify miR-193a binding to the potential binding site of PSEN1, the binding sequences were mutated from GGCCAGU to CCGGUCA, and then the luciferase activity was calculated (Figure 5(a)). The luciferase reporter assay elucidated that miR-193a decreased (*P* < 0.05) the luciferase activity of HGC-27 cells transfected with wild-type PSEN1 3′-UTR, while it did not alter (*P* > 0.05) the luciferase activity of cells transfected with mutant PSEN1 3′-UTR (Figure 5(b)). PSEN1 mRNA levels were evaluated after transfection of miR-193a mimic or miR-193a inhibitor in HGC-27 cells. As expected, overexpression of miR-193a inhibited (*P* < 0.05) the mRNA level of PSEN1, while knockdown of miR-193a enhanced (*P* < 0.05) PSEN1 expression in HGC-27 cells (Figure 5(c)). All the results indicated that miR-193a regulated the expression of PSEN1 in gastric cancer cells HGC-27.

### 3.6. PSEN1 Reverses the Roles of miR-193a in Gastric Cancer Development

**Figure 6:**
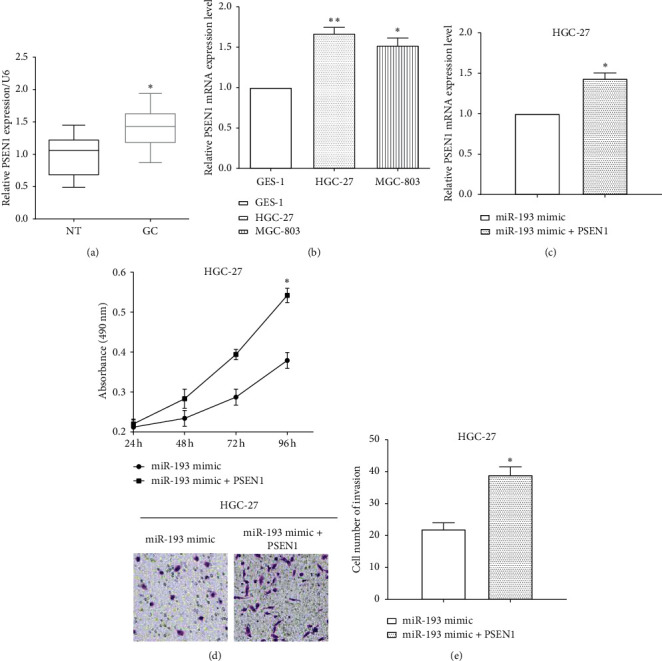
PSEN1 reverses the roles of miR-193a on cell proliferation and invasion. (a) PSEN1 was upregulated in gastric cancer tissues compared to peritumoral normal tissues. (b) PSEN1 expression was higher in HGC-27 and MGC-803 cells than that in GES-1 cells. (c) PSEN1 overexpression plasmid was transfected in miR-193a overexpressed HGC-27 cells. (d) Upregulation of PSEN1 enhanced cell proliferation in miR-193a mimic-transfected HGC-27 cells. (e) PSEN1 reversed partial roles of miR-193a on cell invasion. ^*∗*^*P* < 0.05; ^*∗∗*^*P* < 0.01.

The expression of PSEN1 was evaluated by RT-qPCR and we found that it was downregulated in peritumoral normal tissues compared to that in gastric cancer tissues (*P* < 0.05) (Figure 6(a)). Similarly, the expression of PSEN1 was higher in HGC-27 (*P* < 0.01) and MGC-803 (*P* < 0.05) cells than that in GES-1 cells (Figure 6(b)).

To investigate the effect of PSEN1 on the suppressive role of miR-193a, PSEN1 overexpression plasmid was transfected in miR-193a overexpressed HGC-27 cells (Figure 6(c)). Upregulation of PSEN1 enhanced cell proliferation and invasion in miR-193a mimic-transfected HGC-27 cells (Figures 6(d) and 6(e)). All the results revealed that PSEN1 partially reversed the roles of miR-193a on cell proliferation and invasion.

## 4. Discussion

Gastric cancer, a kind of gastrointestinal malignancy, is the second leading death cancer, and the early diagnosis is very difficult [1, 2]. Thus, exploring new biomarkers for the early diagnosis and treatment of gastric cancer is urgent.

Micro-RNAs caused mRNA degradation or translational inhibition through binding to target mRNAs 3′-UTR at the posttranscriptional level [21, 22]. Accumulating evidence indicated that multiple miRNAs were involved in tumor prognosis in gastric cancer, including miR-127, miR-575, miR-31, and miR-520c [23–26]. miR-193a acted as a key tumor suppressor in various cancers and played significant roles in tumor growth and metastasis [8, 11]. miR-193a inhibited cell viability, proliferation, and colony formation and induced *G*1 phase arrest in prostate cancer cells [27]. Consistent with all the findings above, we discovered that miR-193a was downregulated in gastric cancer tissues and cells, and downregulation of miR-193a predicted poor 5-year overall survival of gastric cancer patients. miR-193a inhibited tumor growth in vitro and in vivo and suppressed tumor metastasis in gastric cancer cells HGC-27. Pan et al. indicated that miR-193a inhibited cell proliferation and invasion through PI3K/AKT pathway in renal cell carcinoma [28]. Our results are consistent with Pan et al.; miR-193a suppressed the activation of PI3K/AKT pathway in gastric cancer. What is more, we first propose that miR-193a inhibited the epithelial-to-mesenchymal transition (EMT) of gastric cancer cells. However, miR-193a promoted cell proliferation and migration in esophageal squamous cell carcinoma [29]; therefore, we conjectured that miR-193a might have tissue specificity.

PSEN1 is a primary component of the *γ*-secretase complex, which is mainly related to Alzheimer's disease [14, 15]. PSEN1 downregulation caused decreased neuronal survival and protected neurons from glucose deprivation-induced death [30]. Even in gastric cancer, Li et al. indicated that PSEN1 enhanced carcinogenesis and metastasis [31]. PSEN1 is a direct target gene of miR-193a, and PSEN1 promoted cell apoptosis and enhanced the sensitization to a drug in bladder cancer [32]. Consistent with Meng et al. [18, 32], we found that PSEN1 was a target of miR-193a and its expression was mediated by miR-193a in gastric cancer cells HGC-27. PSEN1 partially reversed the functions of miR-193a in cell proliferation and invasion in HGC-27 cells. The shortcoming of our study is that it has not further explored the mechanism of the miRNA/PSEN1/PI3K/Akt axis in gastric cancer.

## 5. Conclusions

miR-193a inhibits gastric cancer cell proliferation, invasion, and the epithelial-to-mesenchymal transition and suppresses the activation of the PI3K/Akt signaling pathway. This study evaluates the therapeutic potential of miR-193a in gastric cancer and provides a new target for the prevention and treatment of gastric cancer.

## Data Availability

The datasets used and/or analyzed during the present study are available from the corresponding author on reasonable request.
